# Erchen Plus Huiyanzhuyu Decoction Inhibits the Growth of Laryngeal Carcinoma in a Mouse Model of Phlegm-Coagulation-Blood-Stasis Syndrome via the STAT3/Cyclin D1 Pathway

**DOI:** 10.1155/2020/2803496

**Published:** 2020-04-22

**Authors:** Xi Tan, Qiulan Luo, Shiqing Zhou, Wei Huang, Xiaocong Feng, Wenyong Chen, Chaojie Yang, Yunying Li

**Affiliations:** ^1^Otorhinolaryngology Department, The Second Affiliated Hospital of Guangzhou University of Chinese Medicine, Guangzhou 510120, China; ^2^Otorhinolaryngology Department, Guangdong Provincial Hospital of Chinese Medicine, Guangzhou 510120, China; ^3^The Second Clinical College of Guangzhou University of Chinese Medicine, Guangzhou 510120, China

## Abstract

Erchen plus Huiyanzhuyu decoction (EHD), a Chinese herbal medicine (CHM) formula that consists of Erchen decoction and Huiyanzhuyu decoction, has achieved satisfactory results in the clinic. The main function of EHD is to remove phlegm and blood stasis, and EHD is suitable for phlegm-coagulation-blood-stasis (PCBS) syndrome in laryngeal cancer (LC). In this study, a xenograft mouse model of LC with PCBS syndrome was constructed by feeding a high-fat diet, swimming in ice water, and subcutaneously injecting epinephrine hydrochloride for 2 weeks. Based on the successful Chinese medicine syndrome model, Hep2-luciferase-GFP cells were injected subcutaneously under the armpit of the right upper limb in mice to form tumours. A mouse model of LC with PCBS syndrome was established via heterotopic transplantation. Then, the mice received intragastric administration of different concentrations of EHD daily, and cisplatin (DDP) was intraperitoneally injected every week for 21 days. Tumour fluorescence in mice was measured with a living animal imager on days 7, 14, 21, and 28 during treatment. The results of this experiment confirmed that a mouse model of Chinese medicine syndrome was successfully constructed. Moreover, EHD slowed the growth of xenograft tumours in nude mice; decreased the expression levels of STAT3, p-STAT3, and cyclin D1; and upregulated the expression level of P27. In brief, EHD inhibited laryngeal tumour growth in a xenograft mouse model of PCBS syndrome and regulated the STAT3/cyclin D1 signalling pathway. This study was the first to construct a Chinese medicine xenograft mouse model of LC with PCBS syndrome; in addition, this study clarified that EHD regulated the STAT3/cyclin D1 signalling pathway to inhibit the growth of LC and that EHD may be a promising novel therapeutic compound for the treatment of patients with LC.

## 1. Introduction

Laryngeal squamous cell carcinoma (LSCC) is a common malignant tumour of head and neck cancer, accounting for 5.7%–7.6% of all malignancies, and there is an upward trend in the incidence rate of this disease [1, 2]. The mortality and recurrence rates are still high after surgery, radiotherapy, and chemotherapy. LSCC has already threatened people's lives and health [3, 4]. Therefore, new prevention and control therapies are urgently needed.

Chinese medicine formulae are composed of multidrug and multicomponent formulae based on Chinese medicine principles and are believed to target multiple pathways to treat cancer. Phlegm-coagulation-blood-stasis (PCBS) is the most basic syndrome type of laryngeal cancer (LC). The main effect of Erchen plus Huiyanzhuyu decoction (EHD) is to remove phlegm and blood stasis, and EHD has achieved satisfactory clinical results. It consists of two classic decoctions, Erchen decoction (ECD) and Huiyanzhuyu decoction (HYZYD). ECD originated from a book titled *Prescriptions from the Great Peace Imperial Grace Pharmacy* [5] and is widely used for the treatment of various cancers, including head and neck cancers [6, 7], lung tumours [8–10], and gastrointestinal carcinomas [11]. HYZYD was first introduced for laryngeal diseases by the renowned physician Wang Qingren in the Qing dynasty, and HYZYD recorded in his classic medicine book *Correction of Errors in Medical Classics* [12]. We previously found that modified EHD could relieve symptoms, improve recovery, and reduce the recurrence of precancerous lesions of LC diseases, including laryngeal papilloma and laryngeal leukoplakia [13].

It has been suggested that the occurrence and development of LSCC are regulated by many genes. Signal transducer and activator of transcription 3 (STAT3) is highly expressed in LC. It plays an important role in the occurrence, development, metastasis, and prognosis of LC. Studies have shown that the persistent activation of STAT3 is closely related to the malignant transformation of tumours [14]. Selective knockout of the STAT3 gene will block the transduction of related signalling pathways in cancer treatment [15, 16]. STAT3, which acts on nuclear DNA, is activated by extracellular cytokines, growth factors, and other polypeptide ligands [17].

STAT3 does not directly induce tumourigenesis but influences the progression of cancer by regulating its downstream target genes. STAT3 can regulate the growth cycle of cancer cells by affecting the expression of cyclin D1 and P27 [18]. Cyclin D1 and P27 are closely related to pathophysiological processes such as cell proliferation and apoptosis inhibition [19].

Additionally, our in vitro study demonstrated that EHD could decrease the protein expression levels of STAT3 and p-STAT3 and regulate the cell cycle and induce apoptosis in Hep2 and Tu212 cells of the LC cell line. An animal model of the heterotopic transplantation of LC with PCBS was successfully established in mice to observe the efficacy of EHD in controlling cancer progression, improving quality of life, and reducing the side effects of chemotherapy in vivo. The STAT3/cyclin D1 pathway can be considered a practical target for therapeutic intervention in LC. The present study explored how EHD extract regulated STAT3 and downstream target genes to inhibit tumour growth.

## 2. Materials and Methods

### 2.1. Preparation of Freeze-Dried Powder of EHD Water Extract

EHD is composed of 15 traditional Chinese medicines, including Ban-Xia (Pinellia Rhizoma, 10.7%), Ju-Hong (Citri Exocarpium Rubrum, 10.7%), Fu-Ling (Poria, 6.4%), Sheng-Jiang (Zingiberis Rhizoma Recens, 7.1%), Wu-Mei (Fructus Mume, 2.1%), Tao-Ren (Persicae Semen, 10.7%), Hong-Hua (Carthami Flos, 10.7%), Gan-Cao (Glycyrrhizae Radix et Rhizoma, 9.6%), Jie-Geng (Platycodonis Radix, 6.4%), Sheng-Di-Huang (Rehmanniae Radix, 8.5%), Dang-Gui (Angelicae Sinensis Radix, 4.3%), Xuan-Shen (Scrophulariae Radix 2.1%), Chai-Hu (Bupleuri Radix, 2.1%), Zhi-Ke (Fructus Aurantii, 4.3%), and Chi-Shao (Paeoniae Radix Rubra, 4.3%). All herbs were purchased from KANGMEI Pharmaceutical Co., Ltd. (Guangzhou, China). The mixture (140.5 g) was soaked in deionized water for 30 min before boiling twice at 100°C with 1.5 litres of deionized water for 2 h. The filtrates were concentrated with a rotary evaporator and dried by lyophilization to obtain the dried powder. The extract was stored at 4°C. Simultaneously, EHD was analysed on an Agilent 1260 LC HPLC system (diode array, chemiluminescence, CA, USA) using an Agilent 5 TC-C18 column (260 *∗* 4.6 mm, CA, USA). The mobile phase consisted of acetonitrile and water containing 0.05% phosphoric acid at a column temperature of 30°C and at a flow rate of 1 ml/min with an injection volume of 10 *μ*l. The wavelength was set at 254 nm for detection.

### 2.2. Instruments and Reagents

A small animal in vivo imaging system (IVIS Lumina XR, CA, USA), a Rado automatic coagulation analyser (RAC-030, Shanghai, China), a biochemical analyser (Cobas 8000, Roche, Basel, Switzerland), an automated cell counter (Countstar, IC-1000, USA), Applied Biosystems ViiA™ 7 Dx (Thermo Scientific, Ma, USA), a high-sensitivity ChemiDoc Touch-1 Imaging System (Bio-Rad, CA, USA), a fully automatic inverted fluorescence microscopic analysis system (Nikon, Japan), an enzyme labelling instrument (VICTOR X5, PerkinElmer, USA), a gas anaesthesia system (Ryward, CA, USA), a luciferase in vivo imaging substrate (Promega Biotechnology Co., Ltd., Beijing, China), epinephrine hydrochloride (Lishuan, Chinese pharmaceutical standard no. H42021700), cisplatin (DDP) injection (Jiangsu Hausen Pharmaceutical Co., Ltd., Chinese pharmaceutical standard no. 20040813), a chemiluminescence imaging system (Bio-Rad, CA, USA), monoclonal antibodies against STAT3, p-STAT3, cyclin D1, P27, and GAPDH (Cell Signalling Technology, Inc., Danvers, MA, USA), PCR primers (Shanghai Zhenzhi Biology Co., Ltd., Shanghai, China), phenol-free red RPMI, pancreatin, PBS (Thermo Scientific, MA, USA), serum (Gibco, Australia), a thermometer, and a 1 ml syringe were used among other materials.

### 2.3. Cell Transfection Experiment

The Hep2 cell line was provided by the Biological Laboratory of the Second Affiliated Hospital of Sun Yat-sen University. Virus particles were packed using HEK-293T cells and plasmid transfection. Then, the recombinants were transfected into Hep2 cells, demonstrating that the genes were expressed in the host by an immunofluorescence assay. Hep2-luciferase-GFP cells were cultured and passaged in 1640 RPMI medium containing 10% FBS, penicillin (100 U/ml), and streptomycin (100 mg/ml) (Invitrogen, Carlsbad, CA, USA) at 37°C in a 5% CO_2_ humidified atmosphere.

### 2.4. Animal Experiments

The experiment was carried on BALB/c nude mice, 16.5 ± 1.0 g, females, provided by Beijing Weitong Lihua Experimental Animal Technology Co., Ltd., certificate no. SCXK (Beijing) 2012-0001. The experiment began after 3 days of animal quarantine. Animals were raised in the SPF environment in separate cages, with 6 animals per cage. The animals were randomly divided into a blank group (*n* = 12) and a model group (*n* = 48). All the experiments and animal care procedures were approved by the ethics committee of Guangdong Chinese Medicine Hospital (no. 2018013).

The animal model of PCBS syndrome was established by high-fat diet feeding, ice water swimming, and subcutaneously injecting epinephrine hydrochloride. The mice were given free access to a high-fat diet, the composition of which included sucrose (20%), lard (15%), cholesterol (1.2%), sodium cholate (0.2%), casein (10%), calcium hydrogen phosphate (0.6%), stone powder (0.4%), premix (0.4%), and basic feed (52.2%). The diet was provided by Guangdong Medical Laboratory. Mice swam in ice water at temperatures ranging from 0 to 4°C. The nude mice were lifted out from the water tank when they were unable to raise their heads and sank into the water, and then dried with a towel. After 30 min of swimming, epinephrine hydrochloride (0.2 mg/20 g, 0.9% saline dilution) was injected subcutaneously into the abdomen. Control nude mice were fed a normal diet daily, swam in normal temperature water, and were subcutaneously injected with normal saline into the abdomen. The symptoms and signs of the mice were observed, and the weights and the amounts of drinking water and food consumed by the mice were recorded every other day during the syndrome modelling period. Two weeks later, the blood was taken from the eyeball under gas anaesthesia, and the blood lipids and coagulation index of the whole blood supernatant were detected.

Successful mouse models of PCBS syndrome were randomly divided into 5 groups (seven mice per group) [20, 21]. Cells were collected and suspended in phenol-free red medium containing 1% serum. Mice were subcutaneously injected below the armpit of the right forelimb (2.0 × 10^6^ cells per animal). When the tumours reached an average size of 100 mm^3^, the mice received an intragastric administration of different concentrations of EHD every day. DDP was given as an intraperitoneal injection every week. The dosage of the medium of EHD given to the mice was based on the daily equivalent dose given to humans. Compared with the medium-dose group, the high-dose group received a double dose, and the low-dose group received half of the dose. DDP was also applied based on the daily equivalent dose given to humans. In the clinical practice of CHM, EHD is usually prescribed at a daily dose of 140.5 g of raw herbal material. When this human dose was converted into an animal dose (a person weighing 60 kg and a conversion factor of 12.33 between humans and mice), it was equivalent to the middle dose (28.87 g/kg) used in this study. Therefore, EHD was administered every day at doses of 14.44, 28.87, and 57.74 g/kg.

Tumour fluorescence in mice was measured with a living animal imager on days 7, 14, 21, and 28 during treatment. The luciferase substrates of fireflies were dissolved in PBS and injected intraperitoneally at a dose of 150 mg/kg body weight for 10 min before animal tumours were detected via a fluorescence imaging system. A region of interest (ROI) value was obtained from the tumour location in each mouse, and the number of photon signals from the mouse body surface area was calculated (per/s/cm^2^/sr). A red signal indicates high intensity, and a purple signal indicates low intensity. Finally, the data were collected for analysis.

### 2.5. Western Blot Analysis

Tumour tissue was shredded and lysed with RIPA assay buffer (Cell Signalling Technology, Inc., Shanghai, China) on ice for 30 min. The protein concentration was determined using a bicinchoninic acid protein assay kit (Thermo Fisher Scientific, Inc., CA, USA). Equal protein (40 *μ*g) per lysate was resolved on a Tris-glycine gel, transferred onto a nitrocellulose membrane, and blocked for 1 h with 5% non-fat milk in TBS containing 1% Tween-20. Membranes were incubated with the desired primary antibody (STAT3, p-STAT3, P27, cyclin D1, or GAPDH; 1 : 1,000 dilution) overnight at 4°C and then with the appropriate secondary antibody (1 : 3,000) for 1 h. Densitometric analysis was performed using a chemiluminescence imaging system. GAPDH was used as a control for each sample.

### 2.6. Reverse Transcription Quantitative Polymerase Chain Reaction (RT-PCR) Analysis

Total RNA was extracted with a tissue total RNA extraction kit (Vazyme, RC101, Nanjing, China). The RNA purity and concentration were detected using a spectrophotometer (NanoDrop 2000c, Thermo Fisher Scientific, CA, USA). cDNA was synthesized from 1 *μ*g of oligo (dT)-primed RNA obtained from each sample with reverse transcription kit (Vazyme, Nanjing, China) according to the manufacturer's instructions. Then, 1 *μ*g of cDNA was used to determine the amount of STAT3, cyclin D1, and P27 transcripts via polymerase chain reaction. GAPDH was used as an internal control. The sequences of the primers used for GAPDH, STAT3, cyclin D1, and P27 are as follows: STAT3 (20 bp), forward: 5′-CAA GGG CTT CTC CTT CTG GG-3′ and reverse: 5′-GGA TCT GGG TCT TAC CGC TG-3′; cyclin D1 (20 bp), forward: 5′-GAG GAA GAG GAG GAG GAG GA-3′ and reverse: 5′-GAG ATG GAA GGG GGA AAG AG-3′; P27 (20 bp), forward: 5′-CGA TAG CTG TGT GCA AAG TAA CT-3′ and reverse: 5′-CCA TCT GCT GAG TGC TTT CTG-3′; and GAPDH (20 bp), forward: 5′-ACT CCT CCA CCT TTG ACG CT-3′ and reverse: 5′-GGT CTC TCT CTT CCT CTT GTG C-3′. We used the 2^−ΔΔCt^ method to determine the relative mRNA expression levels in at least three independent experiments.

### 2.7. Histological Haematoxylin-Eosin (HE) Staining and Immuno-Histochemical Analysis

Tumour specimens and kidney tissues were harvested and immersed in 4% paraformaldehyde fixation for 20 h. All the embedded samples were cut into 5 *μ*m sections. Kidney tissues were prepared for HE staining according to standard protocols, and tumour specimens were prepared for immunohistochemistry. Sections for immunohistochemistry were dewaxed, antigen-repaired and sealed, and incubated at 4°C overnight with anti-STAT3 (1 : 1000) and anti-cyclin D1 (1 : 50) antibodies. After the slide was washed three times, a DAB reagent kit (Solarbio, Guangzhou Weijia Biological Co., Ltd., China) was used for staining, and haematoxylin was used for counterstaining. We recorded the results on a positive imaging microscope (Olympus BX53, Leica, Germany). The data were analysed with Image-Pro Plus IPP6.0.

### 2.8. Statistical Analysis

The analyses and drawings of all experimental data were carried out with GraphPad Prism 6.0 software. The measurement data are expressed as the mean ± standard deviation (SD) of at least three independent experiments. When two comparisons were made and the data followed normal distribution, the parameter *T* test was used. An unpaired *t*-test was used in the presence of homogeneity of variance, and in the presence of inhomogeneity of variance, a Welch's correction unpaired *t*-test was used. *p* value < 0.05 was considered statistically significant.

## 3. Results

### 3.1. Identification of the Main Active Compounds of EHD

**Figure 1:**
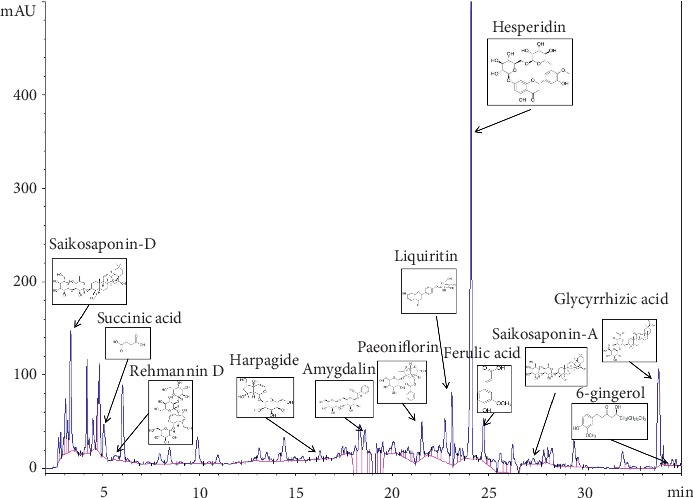
HPLC chromatogram for EHD.

We aimed to guarantee the repeatability of future experiments by ensuring the quality and stability of EHD. The active components of EHD and their concentrations were analysed by HPLC. As shown in Figure 1, compared to the standards, we identified 12 active compounds, namely, paeoniflorin, amygdalin, harpagide, glycyrrhizic acid, liquiritin, saikosaponin-A, saikosaponin-D, ferulic acid, succinic acid, rehmannin D, hesperidin, and 6-gingerol. These identified active ingredients accounted for more than 30% of the total. The results were analysed on an Agilent 1260 LC HPLC system using an Agilent 5 TC-C18 column (260 × 4.6 mm). The mobile phase consisted of acetonitrile and water containing 0.05% phosphoric acid at a column temperature of 30°C and at a flow rate of 1 ml/min with an injection volume of 10 *μ*l. The wavelength was set at 254 nm for detection.

### 3.2. Observation of the Body Weight and Diet and Water Intake during the Establishment of the Syndrome Model

**Figure 2:**
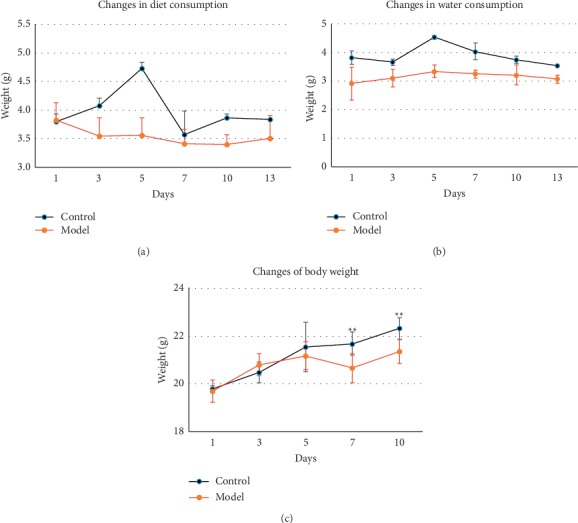
Changes in the diet, water intake, and body weight over the 14 days needed to establish PCBS syndrome in nude mice. (a) Dietary changes during syndrome modelling; (b) changes in water intake during syndrome modelling; (c) changes in body weight during syndrome modelling. Values in the line chart represent the mean ± SD (*n* = 8). ^*∗∗*^*p* < 0.01 vs. control.

The body weights, dietary intake, and water intake in mice were recorded during the modelling period. The dietary intake and water intake of the general situation in the model group were less than those in the blank group. However, there was no significant difference between the two groups. After one week, the average weight of mice in the blank group increased faster than that of the mice in the model group, and the difference between the two groups was statistically significant (*p* < 0.01) (Figure 2).

### 3.3. A Nude Mouse Model of PCBS Syndrome Was Successfully Established

**Figure 3:**
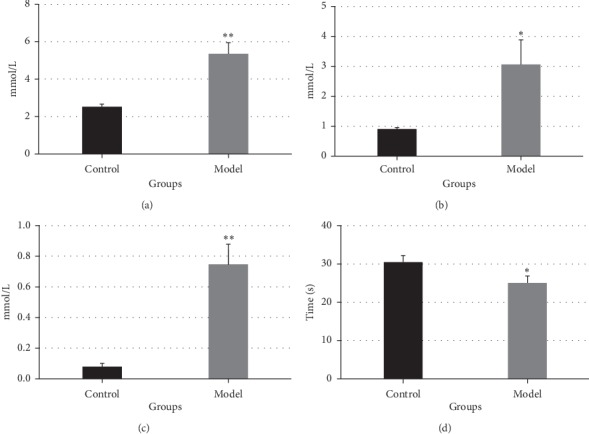
Evaluation of blood lipids and the coagulation function in the mouse model of PCBS syndrome. Serum total cholesterol (a) (TC), non-high-density lipoprotein cholesterol (b) (non-HDL-C), low-density lipoprotein cholesterol (c) (LDL-C), and activated partial thrombin time (d) (aPTT) (*n* = 3). ^∗^*p* < 0.05, ^∗∗^*p* < 0.01 vs. control.

The nude mouse model of PCBS syndrome was established by feeding the mice a high-fat diet, having the mice swim in ice water, and subcutaneously injecting epinephrine hydrochloride for 3 weeks. As shown in Figure 3, total cholesterol (TC), non-high-density lipoprotein cholesterol (non-HDL-C), and low-density lipoprotein cholesterol (LDL-C) in the sera of the model group mice were significantly increased compared to those in the sera of the control group mice. Furthermore, the activated partial prothrombin time (aPTT) in the sera was shorter in the model group mice than in the control group mice, and the difference between the two groups was statistically significant. These results suggest changes in blood lipids and coagulation function in the mouse model.

### 3.4. EHD Slows the Growth of Xenograft Tumours in Nude Mice

**Figure 4:**
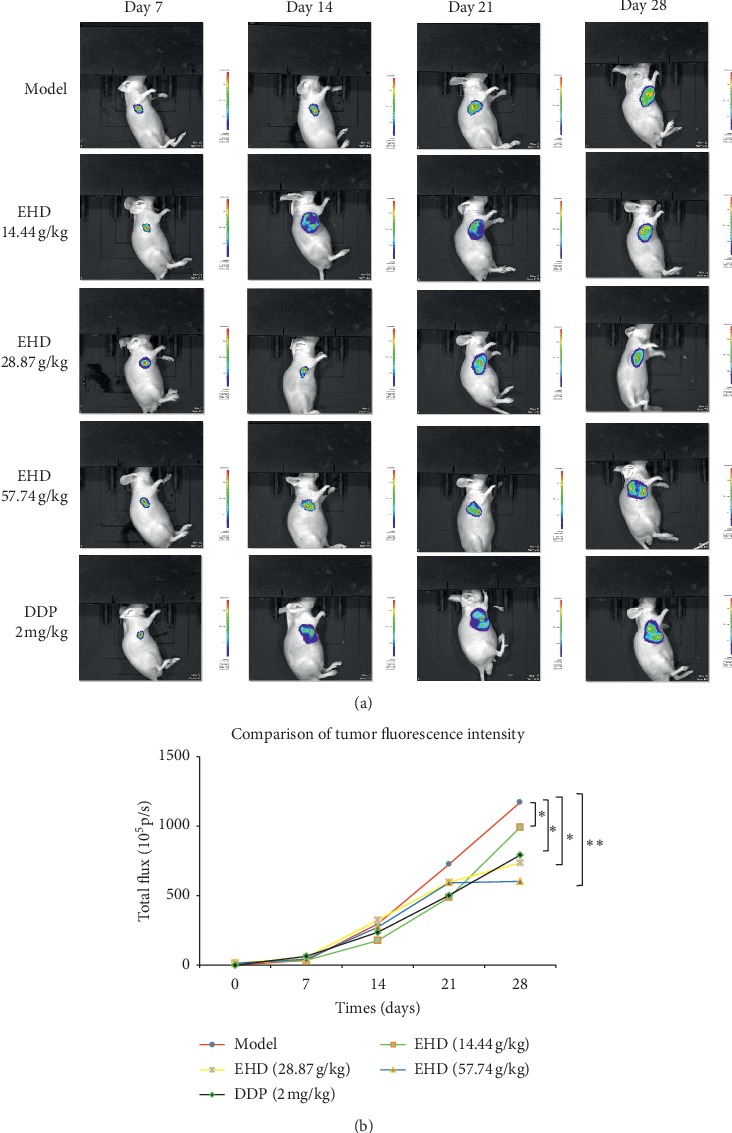
EHD slows the growth of LC in vivo. The fluorescence intensities of tumours in the different groups were recorded every other week with a living animal imager system. The tumour fluorescence intensities were calculated at different stages of treatment. Data are shown as the mean ± SD. ^*∗*^*p* < 0.05, ^*∗∗*^*p* < 0.01, ^*∗∗∗*^*p* < 0.001 vs. model.

To elucidate the antitumour effect of EHD, the drug was administered continuously for 3 weeks after the tumour cells were implanted into the animals for a week. The fluorescence intensities of the tumours in different groups were recorded every other week with a living animal imager system. The fluorescence intensity of the different groups of xenograft tumours was compared at the final stage. As shown in Figure 4, different concentrations of EHD could delay the growth of tumours, and so did DDP. The difference between the two groups was statistically significant.

### 3.5. EHD Decreases the Protein Expression of STAT3, p-STAT3, and Cyclin D1 and Increases the Expression of P27

**Figure 5:**
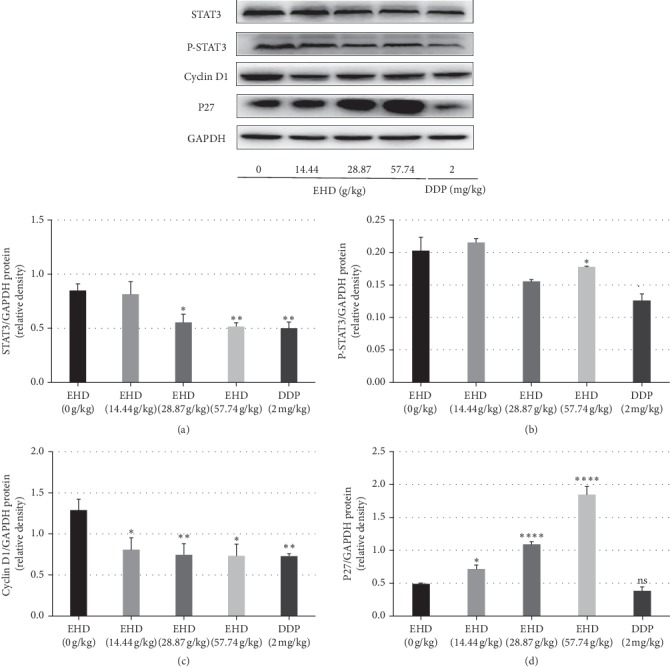
Effects of EHD on the STAT3/cyclin D1 pathway in the tumour tissues of nude mice with LC and PCBS syndrome. Western blot analysis of the levels of STAT3, p-STAT3, cyclin D1, and P27 in nude mice treated with 14.44, 28.87, or 57.74 g/kg EHD and 2 mg/kg DDP for 3 weeks. GAPDH was used to ensure equal loading of proteins in each lane. All data are presented as the mean ± SD of three independent experiments. (a) Treatment with 28.87 and 57.74 g/kg EHD and 2 mg/kg DDP inhibited STAT3 protein expression. (b) Treatment with 28.87 and 57.74 g/kg EHD and 2 mg/kg DDP also decreased p-STAT3 protein expression. (c) Different concentrations of EHD and 2 mg/kg DDP downregulated cyclin D1 protein expression. (d) EHD upregulated the P27 protein levels in a dose-dependent manner, but 2 mg/kg DDP had no effect on the P27 protein levels. ^*∗*^*p* < 0.05, ^*∗∗*^*p* < 0.01, and ^*∗∗∗*^*p* < 0.0001 vs. the control group (0 g/kg EHD).

To elucidate the mechanisms of action of EHD in LC treatment, the protein levels of STAT3/p-STAT3, cyclin D1, and p27 were determined. We used Western blotting to investigate whether EHD affects the STAT3/cyclin D1 pathway in a nude mouse model. As shown in Figure 5, at concentrations of 28.87 and 57.74 g/kg, EHD attenuated the protein expression levels of STAT3 and p-STAT3 in laryngeal carcinoma tumour tissue in nude mice. Compared with the control group, the difference was statistically significant. EHD decreased cyclin D1 protein expression. DDP also decreased STAT3, p-STAT3, and cyclin D1 protein expression levels. Moreover, EHD increased P27 protein levels in a dose-dependent manner, whereas DDP did not.

### 3.6. EHD Regulates the STAT3/Cyclin D1 Signalling Pathway at the Genetic Level

**Figure 6:**
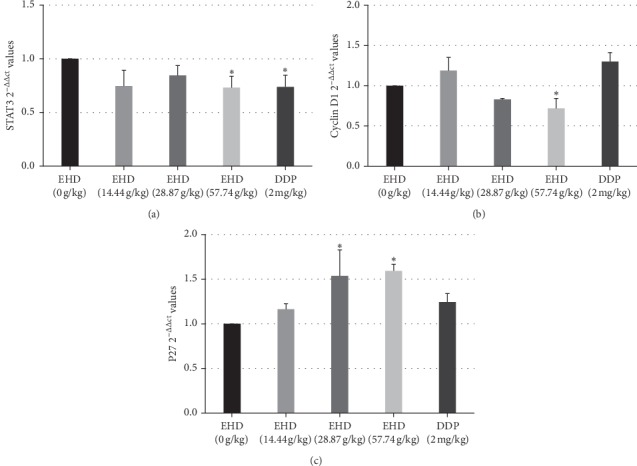
mRNA expression levels of STAT3, cyclin D1, and P27 in LC tumour tissues from nude mice with PCBS syndrome were assessed by RT-PCR. (a–c) The levels of STAT3, cyclin D1, and P27 in nude mice treated with 14.44, 28.87, or 57.74 g/kg EHD and 2 mg/kg DDP for 3 weeks. GAPDH is a housekeeping gene. All data are presented as the mean ± SD of three independent experiments. ^*∗*^*p* < 0.05 vs. the control group (0 g/kg EHD).

We used RT-PCR to explore whether EHD affected the STAT3/cyclin D1 signalling pathway at the genetic level. As shown in Figure 6, the experimental results showed that a high dose (57.74 g/kg) of EHD could attenuate the mRNA levels of STAT3 and cyclin D1. Compared with the control group, the difference was statistically significant. In addition, DDP downregulated the STAT3 mRNA level but had no effect on the cyclin D1 mRNA level. Moreover, EHD could upregulate the P27 mRNA level, whereas DDP had no effect on P27 mRNA.

### 3.7. Immunohistochemical Experiments Also Demonstrated That EHD Downregulated the STAT3 and Cyclin D1 Protein Expression Levels

**Figure 7:**
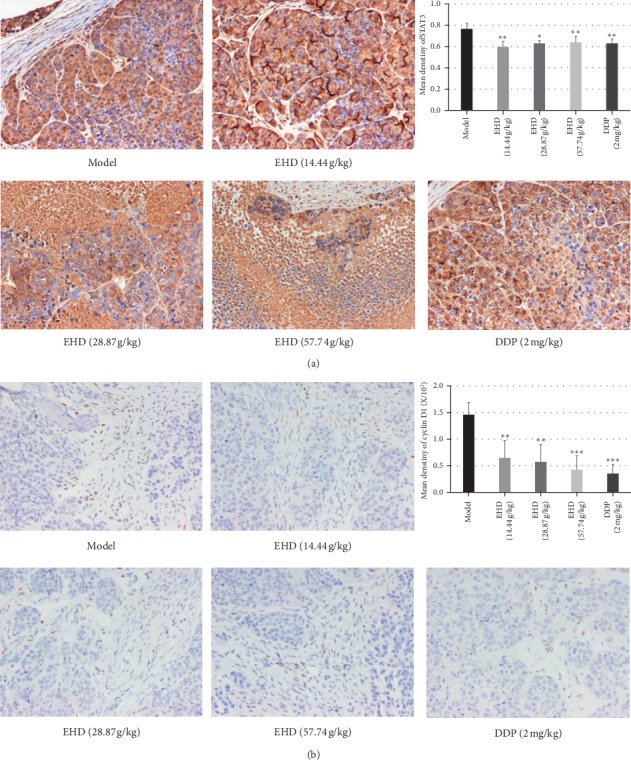
Detection of cyclin D1 and STAT3 levels in different groups by immunohistochemical analyses. (a and b) Immunohistochemistry for STAT3 and cyclin D1 on the tumour tissues in different groups of nude mice. Magnification, x 400. Positive cells were stained brown. The results showed that EHD could significantly reduce the expression of cyclin D1 and STAT3. ^*∗*^*p* < 0.05, ^*∗∗*^*p* < 0.01, and ^*∗∗∗*^*p* < 0.001 vs. Mo. All data are presented as the mean ± SD and were obtained by choosing 3 fields of view for each section, 2 sections in total.

To further elucidate the effect of EHD on the STAT3/cyclin D1 signalling pathway, we analysed the STAT3 and cyclin D1 levels in xenograft tumours in nude mice by immunohistochemistry analyses. The results confirmed that EHD could significantly reduce the expression of cyclin D1 and STAT3 (Figure 7). Tumour tissues were removed from nude mice on the 21st day after the administration of EHD and DDP. Image-Pro Plus 6.0 software was applied to analyse the average optical density of the images. The results showed that EHD could significantly reduce the expression of cyclin D1 and STAT3.

### 3.8. EHD Has Lower Toxicity and Fewer Side Effects Than DDP on the Kidneys In Vivo

**Figure 8:**
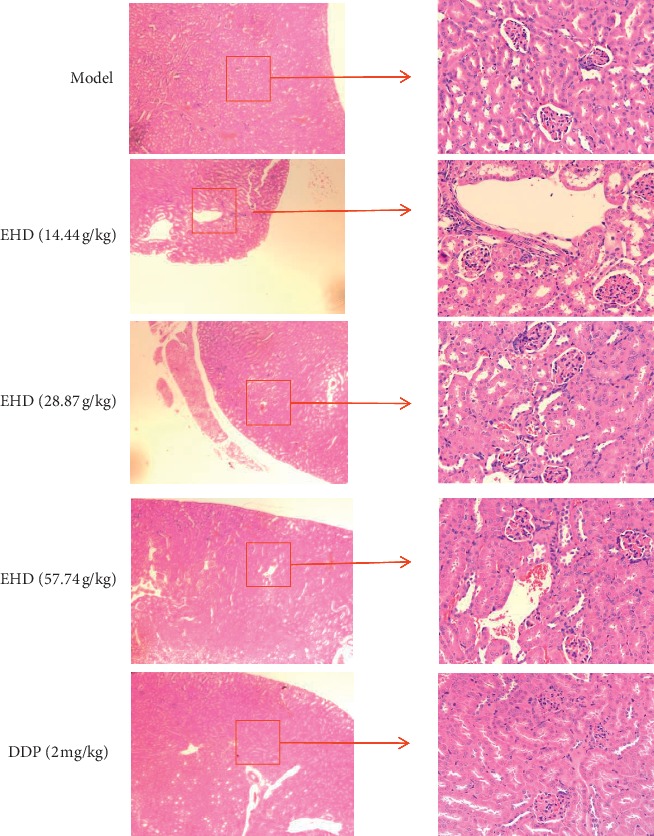
Effect of EHD on the renal structure in mice by HE staining. EHD had less toxicity and fewer side effects on the kidneys than DDP in vivo. The kidneys were removed from nude mice on the 21st day after administration. The kidneys of mice in the Western medicine group showed tubular cell oedema and tissue gap narrowing, while the kidneys of mice in the EHD group were not damaged. Magnification, 400x.

To observe the toxicity and side effects of EHD, we harvested the kidneys from nude mice 21 days after the administration of EHD and DDP and carried out HE staining. As shown in Figure 8, the renal tubular cells exhibited oedema, a wide membrane, a narrow tissue space, and mesangial matrix accumulation in the Western medicine (DDP) group. However, there was no destruction of the renal tissue structure, and matrix hyperplasia decreased significantly in the EHD group compared with that in the DDP group.

### 3.9. Changes in the Appearance of the Tongue, Symptoms, and Signs during the Establishment of the Mouse Model of PCBS Syndrome

**Figure 9:**
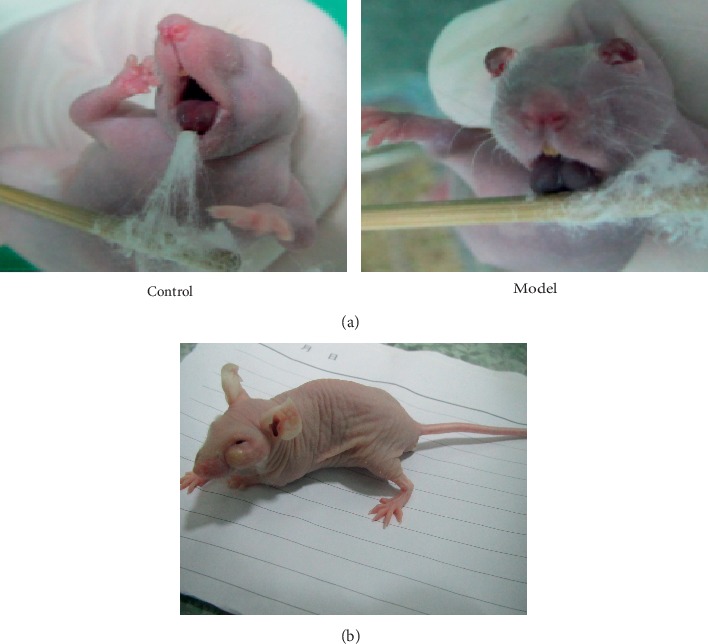
Changes in the appearance of the tongue, symptoms, and signs in the mouse model of PCBS syndrome. (a) The tongue was dark and dry in the mice with PCBS syndrome, but the tongue was ruddy in the control mice. (b) One mouse in the model group developed a spontaneous tumour near the left eye during the establishment of a mouse model of PCBS syndrome.

Two weeks after establishment of the mouse model of PCBS syndrome, the tongues of the mice were observed and compared. As shown in Figure 9, in the control group, the tongue was ruddy and exhibited signs of ecchymosis, but in the model group, the tongue was dark and dry. Surprisingly, one mouse in the model group developed a spontaneous tumour near the left eye while the mouse model of PCBS syndrome was being established. We also observed that, in the control group, the skin colour was light red and warm to the touch, and the stool was normal. However, in the model group, the skin colour was purple and dark, the temperature of the skin was low, and the stool was sticky and not shaped.

## 4. Discussion

Among all head and neck malignant tumours, LC has the second highest incidence worldwide [22]. Approximately 40,000 individuals in the USA are anticipated to develop LC annually (3% of all US cancer cases); of these, 11,000 will die (2% of all US cancer mortalities) [23]. In 2006, LC accounted for 9510 incident cases and 3740 deaths, indicating a high mortality for such a tumour [24]. Chinese medicine offers great potential for enhancing current treatments for LC and reducing the side effects of chemotherapy or radiotherapy. For example, cucurbitacin B, which is extracted from natural herbal agents, has been shown to enhance the chemosensitivity of LSCC cells to DDP by inhibiting the STAT3 pathway [25]. Ying et al. claimed that, by inhibiting AKT/mTOR signalling, pterostilbene decreases the proliferation, apoptosis, and invasiveness of LSCC cells [26]. Zhang Sen et al. demonstrated that monomers derived from CHM have also shown encouraging kidney protective effects and have reduced the toxicity associated with chemotherapy [27, 28].

After years of clinical experience, our team found that “PCBS syndrome” is the most basic syndrome type of LC. Based on the Chinese medicine principle of “promoting blood circulation for removing blood stasis, resolving toxins, and disinhibiting the throat,” “drying dampness and resolving phlegm,” and clinical experience, EHD was formulated. It is mainly composed of two classic formulations of Chinese medicine, namely, ECD and HYZYD. Both have good curative effects in the clinic, including slowing the growth of tumours, improving the quality of life, reducing the side effects of chemotherapy or radiotherapy, and prolonging the survival time in patients with advanced cancer [11, 29, 30]. However, the effect of EHD on LC has not been reported. Therefore, in this study, we explored the anticancer mechanism of EHD.

Ensuring the quality and stability of EHD is aimed at guaranteeing the repeatability of future experiments. Before this experiment, the active components of EHD and their concentrations were analysed by HPLC. As shown in Figure 1, compared to the standards, we identified 12 active compounds, namely, paeoniflorin, amygdalin, harpagide, glycyrrhizic acid, liquiritin, saikosaponin-A, saikosaponin-D, ferulic acid, succinic acid, rehmannin D, hesperidin, and 6-gingerol. The total of the identified active ingredients is more than 30% of the total composition. However, further experiments are needed to analyse and identify these components. It is necessary to detect and analyse the specific anticancer components of EHD.

The syndrome animal model is an important model of experimental research in Chinese medicine, and “treatment according to syndrome differentiation” is one of its major features. Animal models successfully mimicking a syndrome are designed to support the basic theories of Chinese medicine and should be effective in supporting clinical practice. In our study, we first constructed mouse models with PCBS syndrome. In addition to the changes in blood lipids and the coagulation function, there were also changes in the appearance of the tongue, symptoms, and signs in the model group. As shown in Figure 9, in the control group, the skin colour of the mice was light red and warm to the touch, the stool was normal, and the tongue was ruddy with slight ecchymosis. However, in the model group, the skin colour of the mice was purple and dark, the temperature of the skin was low, the stool was sticky and amorphous, and the tongue was dark and dry. Moreover, one mouse in the model group developed a spontaneous tumour near the left eye during the establishment of a mouse model of PCBS syndrome. The changes in the appearance of the tongue, symptoms, and physical signs described above reflect the presence of “phlegm” and “blood stasis” in the model group. These results suggest that we successfully generated an animal model of PCBS syndrome that simulates clinical symptoms.

The STAT family functions in signal transduction and transcriptional activation and plays dual roles in conducting signals and initiating gene transcription in cells [31]. It consists of seven different members: STAT 1, 2, 3, 4, 5A, 5B, and 6 [32]. STATs are expressed in the cytoplasm and become activated through phosphorylation and then enter the nucleus to promote target gene transcription. To date, STAT3 has been found to be constitutively activated in a variety of human malignancies, including squamous cell carcinomas of the head and neck [33].

In this study, EHD not only slowed tumour growth in LC mice with PCBS syndrome but also regulated the STAT3/cyclin D1 signalling pathway. Both the mRNA and protein levels of STAT3 were higher in the mice of xenografts than in the other mice. Because STAT3 is highly expressed in cancer tissues, the level of its activated form (p-STAT3 protein) in tumour tissue is also higher than that in other tissues. This result suggests that STAT3 may be constitutively activated in this mouse model. Moreover, EHD could simultaneously reduce the mRNA and protein levels of STAT3 and the protein level of p-STAT3.

Continuously activated STAT3 is involved in the transcription of cell cycle-related genes, such as cyclin D1 and P27, in LC cells [34]. Activated STAT3 can promote the transition to G1 phase and the process of cancer transformation by upregulating the expression of cyclin D1. The inhibition of STAT3 can downregulate the expression of cyclin D1; thus, cyclin D1 is an important target gene of STAT3 [35]. In our study, the mRNA and protein levels of cyclin D1 were high in the xenograft tumours. When the mice were administered EHD, the mRNA and protein levels of cyclin D1 decreased.

The P27 protein is an important member of the CIP/KIP family of cyclin-dependent inhibitors. The deletion of P27 can induce tumour production, but there are few gene mutations in P27 [36–38]. Therefore, the decrease in P27 expression in tumour tissue is probably not due to a change in its gene level but through a change in its protein level to affect the cell cycle, which leads to tumour occurrence. However, our results showed that EHD could not only increase the protein expression level of P27 but also affect the gene level, while DDP had no effect on the protein expression level of P27.

DDP is one of the main chemotherapeutic drugs for head and neck squamous cell carcinoma [39] and is a non-specific drug targeting cell cycle. The antitumour mechanism of DDP may be through the inhibition of STAT3 signalling pathway [40, 41]. Chemotherapy for LC is associated with a high mortality rate, which is mainly manifested in the toxicity and side effects of drug treatment. In this study, DDP also delayed tumour growth in LC mice with PCBS syndrome and downregulated the STAT3, p-STAT3, and cyclin D1 protein expression levels to some extent. However, DDP always caused severe, toxic side effects in mice. We collected the kidneys of mice that were treated with EHD and DDP for 21 days. We aimed to observe the toxicity and side effects of the drugs. The results showed that EHD did not destroy the kidney tissues of mice; however, DDP caused serious damage to the kidney structure. We observed that the mice in the DDP group had a poor mental state and were thin. This suggests that EHD has lower toxicity and fewer side effects than DDP.

Although EHD can regulate the STAT3/cyclin D1 pathway and delay the growth of LC, it could not completely inhibit the growth of xenograft tumours. To fully exploit the great advantages of Chinese medicine, we can design a combination of EHD and chemotherapeutic agents to reduce toxicity and increase the efficiency of Chinese medicine in the future and provide a promising approach for the treatment of LC.

## Data Availability

The datasets used and/or analysed during the current study are available from the corresponding author upon reasonable request.
